# Pancreatic Islet Transplantation into the Submandibular Gland: Our Experimental Experience and a Review of the Relevant Literature

**DOI:** 10.3390/jcm12113735

**Published:** 2023-05-29

**Authors:** Ibrahim Fathi, Akiko Inagaki, Takehiro Imura, Tarek Koraitim, Masafumi Goto

**Affiliations:** 1 Division of Transplantation and Regenerative Medicine, Tohoku University Graduate School of Medicine, Sendai 980-8575, Japan; i_fathy201010@alexmed.edu.eg (I.F.);; 2 Department of Surgery, Alexandria University, Alexandria 21131, Egypt; 3 Department of Surgery, Tohoku University Graduate School of Medicine, Sendai 980-8575, Japan

**Keywords:** islet transplantation, alternative sites, submandibular gland, intraportal infusion

## Abstract

Pancreatic islet transplantation is a promising therapy for type 1 diabetes. Islet transplantation is clinically performed through intra-portal infusion, which is associated with several drawbacks, including poor engraftment. The histological resemblance between the submandibular gland and the pancreas renders it an attractive alternative site for islet transplantation. In this study, we refined the technique of islet transplantation into the submandibular gland to achieve good morphological features. Then, we transplanted 2600 islet equivalents into the submandibular glands of diabetic Lewis rats. Intra-portal islet transplantation was performed in diabetic rats as a control. Blood glucose levels were followed for 31 days, and an intravenous glucose tolerance test was performed. Immunohistochemistry was used to demonstrate the morphology of transplanted islets. Follow-up after transplantation showed that diabetes was cured in 2/12 rats in the submandibular group in comparison to 4/6 in the control group. The intravenous glucose tolerance test results of the submandibular and intra-portal groups were comparable. Immunohistochemistry showed large islet masses in the submandibular gland in all examined specimens with positive insulin staining. Our results show that submandibular gland tissue can support the islet function and engraftment but with considerable variability. Good morphological features were achieved using our refined technique. However, islet transplantation into rat submandibular glands did not demonstrate a clear advantage over conventional intra-portal transplantation.

## 1. Introduction

Type 1 diabetes mellitus (T1DM) results from the autoimmune destruction of β-cells in the pancreas and requires daily exogenous insulin replacement therapy. In the absence of tight glycemic control, T1DM can result in several complications, including retinopathy, nephropathy, ocular manifestations, and neuropathies. Therefore, pancreatic islet transplantation has been widely investigated, and its ability to offer better glycemic and metabolic control, which cannot be achieved by exogenous insulin therapy, has been demonstrated. At the same time, it alleviates the risk of hypoglycemia unawareness and restores a physiological response to body requirements [1].

Although intra-portal pancreatic islet transplantation has been the most sufficiently characterized method of islet implantation at the experimental and clinical levels, it does not offer an optimal transplantation site, as many of the injected cells are lost during or shortly after implantation [2]. Moreover, the ability of the transplanted cells to maintain tight glycemic control is not sustained over the long term. As a result, several studies have investigated other sites for their potential to maintain pancreatic islet cell function and their feasibility for clinical transplantation [3,4].

Despite its anatomical resemblance to the pancreas, the submandibular gland (SMG) has not been sufficiently investigated as a transplantation site for pancreatic islets. Only a few studies have demonstrated the feasibility of islet transplantation and survival of transplanted cells, and in some instances, the potential of the engrafted cells to reverse diabetes was not investigated [5], or the technique of islet isolation and the number of implanted cells was not optimal when compared to the current methodologies [6].

An ideal transplantation site should allow for minimally invasive islet implantation, ease of monitoring, and the possibility of biopsy and excision, in addition to successful engraftment and long-term maintenance of the islet function [3]. In view of these requirements, the submandibular gland offers an attractive target for investigation. In this study, we optimized the technique of transplantation into the rat submandibular gland and compared it to standard intra-portal transplantation (IPO) based on the glycemic control achieved in diabetic rats.

## 2. Materials and Methods

### 2.1. Animals

Male Lewis rats were purchased from Japan SLC Inc. (Shizuoka, Japan). Male rats were used in this study because they are more susceptible to diabetes induction by STZ compared to female rats. For all experiments, recipients were 8–10 weeks of age, and pancreatic donors were 12–14 weeks of age. The recipient rats were housed separately. All animals had *ad libitum* access to a standard diet and water. The experiments were approved by the local ethics committee (protocol ID: 2017 MdA-294) and performed in accordance with national and institutional regulations. Animals were maintained in a specific pathogen-free environment. All surgeries were performed under anesthesia, and all efforts were made to minimize suffering.

### 2.2. Diabetes Induction

One week before transplantation, male Lewis rats were intravenously injected with streptozotocin (STZ; Sigma–Aldrich, St. Louis, MO, USA) at a dose of 65 mg/kg, according to the previous report [7]. Blood glucose was monitored using a portable glucometer (Freestyle; ABBOTT, Tokyo, Japan). Rats were considered diabetic if the blood glucose level was >400 mg/dL in two consecutive measurements.

### 2.3. Pancreatic Islet Isolation

We isolated pancreatic islets from male Lewis rats as described previously [8]. Briefly, after anesthesia using isoflurane inhalation (Abbott Japan Co., Ltd., Tokyo, Japan), the bile duct was clamped at the papilla of Vater. A total of 10 mL of cold Hanks’ balanced salt solution (HBSS; Sigma–Aldrich) containing 1 mg/mL collagenase (Sigma type V; Sigma Chemicals, St. Louis, MO, USA) was then injected into the common bile duct towards the pancreas. The removed pancreas was incubated in a water bath at 37 °C for 12 min to be digested, and the cell suspension was washed three times in cold HBSS and centrifuged for 1 min. Density-gradient centrifugation using a Histopaque-1119 (Sigma Diagnostics, St. Louis, MO, USA) and Lymphoprep™ (Axis-Shiled, Oslo, Norway) was performed for 10 min to isolate pancreatic islets. The islets were cultured in Roswell Park Memorial Institute Medium 1640 (RPMI-1640, Thermo Fisher Scientific, Waltham, MA, USA) containing 5.5 mmol/L glucose, 10% *v*/*v* fetal bovine serum (Thermo Fisher Scientific), and 1% *v*/*v* penicillin/streptomycin (Thermo Fisher Scientific) at 37 °C in 5% CO_2_ and humidified air.

### 2.4. Islet Transplantation into the Submandibular Gland

After several pilot studies, including direct injection into the parenchyma with a 25-G butterfly needle and subcapsular implantation after making a small incision in the capsule, we adopted the following technique for islet transplantation into the SMG (*n* = 12, SMG group):

One day after isolation, islets were collected for transplantation. Anesthesia was performed as previously described. The hair on the ventral aspect of the neck was shaved using a hair clipper and prepared using 70% *v*/*v* ethanol. A U-shaped incision was made using scissors, and a subplatysmal flap was elevated upwards to expose the submandibular and sublingual glands on both sides. To provide better surgical exposure, the anterior jugular vessels were sometimes severed. The glands were partially mobilized by sharp dissection from the surrounding fibrofatty tissue using scissors, with care taken to avoid injuring the nearby vasculature and gland capsule. Supplementary Figure S1 shows an illustration of the normal SMG anatomy and histology in a Lewis rat.

Then, a 20-G cannula (Terumo, Japan) was introduced into the gland from the distal end along the longitudinal axis of the gland at two sites per gland, the needle was removed, and the cannula was cut to allow the introduction of a smaller 24-G cannula (Terumo) containing the packed islets. The medium was removed from the islets by spinning and aspiration (twice), and the islets were packed into a 24-G cannula by gentle aspiration using a Hamilton syringe (Hamilton Company, Reno, NV, USA) [9]. The cannula was then introduced through the outer cannula, and the islets were slowly implanted as the two cannulas were retracted together (total ~2600 IEQs at four sites, two sites per gland). Gentle compression with a cotton tip was performed at the introduction site, and the glands were moistened with drops of 0.9% *w*/*v* saline. The wound was then closed using 5/0 Nylon sutures. Rats were kept in a warm cage until they made a complete recovery.

### 2.5. Intra-Portal Islet Transplantation

One day after isolation, islets were collected for transplantation. Anesthesia and surgical site preparation were performed as previously described [10]. The hair on the abdomen was shaved using a hair clipper and prepared using 70% *v*/*v* ethanol. A midline laparotomy was performed, and the intestines were gently moved out to the right side on a gauze moistened with saline. The portal vein was exposed, and ~2600 IEQs of islets were suspended in 200 µL of saline. The islets were aspirated using a 25-G butterfly cannula connected to a 1-mL syringe and injected into the portal vein in a total volume of 300 µL. Compression was applied for one minute after needle removal for hemostasis. The wound was then closed in two layers using 4/0 Nylon sutures. Rats were kept in a warm cage until complete recovery (n = 7, IPO group).

### 2.6. Follow-Up after Transplantation

Recipient rats were followed with a measurement of the blood glucose level for 31 days. Blood glucose was monitored using a portable glucometer (Freestyle; ABBOTT, Tokyo, Japan) through a tail vein sample every 3 days for the first 12 days after transplantation and then twice weekly during the follow-up period. Rats were considered cured if their blood glucose level was ≤200 mg/dL in two consecutive measurements. This was followed by an intravenous glucose tolerance test (IVGTT) and SMG excision. Briefly, the wound was re-opened, and the gland was carefully dissected from the nearby vasculature on both sides. Then, the duct and vascular branches to the submandibular and sublingual glands were ligated. The wound was then closed by 5/0 sutures, and the rat was allowed to recover in a warm cage. The blood glucose level was followed after gland excision to confirm the recurrence of diabetes in functional grafts.

### 2.7. IVGTT

The IVGTT was performed as previously described [11,12]. In brief, after fasting for 14 h with *ad libitum* access to water, the body weight and blood glucose level were measured, and 1 g/kg glucose was injected intravenously. The blood glucose level was measured at 5, 10, 20, 30, 60, 90, and 120 min, and the blood glucose curve was generated. The area under the curve (AUC) was then used for comparisons.

### 2.8. Immunohistochemistry

Removed glands were fixed in 4% *v*/*v* paraformaldehyde, dehydrated, and embedded in paraffin blocks. In brief, 4 µm sections were incubated with anti-laminin (Abcam, ab11575, Tokyo, Japan) or rabbit anti-mouse insulin (Abcam, ab181547, Tokyo, Japan). Goat anti-rabbit-HRP (4003; DAKO, Glostrup, Denmark) was used as the secondary antibody for anti-laminin and anti-insulin staining. Either methylene green or hematoxylin was used for nuclear staining. Hematoxylin-eosin (HE) staining was also performed.

### 2.9. Statistical Analyses

The statistical analysis was performed using SPSS version 15.0 for Windows (SPSS, Chicago, IL, USA). A Student’s *t*-test was used to compare the mean AUC values. *p* values of <0.05 were considered statistically significant.

## 3. Results

### 3.1. Implantation Technique

**Figure 1:**
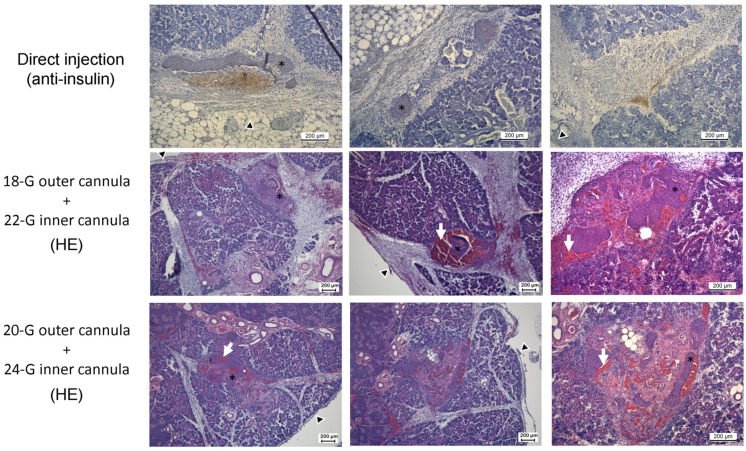
Pilot studies of islet injection into the submandibular gland in Lewis rats (post-transplant day 4 histology). After the direct injection with a 25-G butterfly needle (**uppermost** row), disintegrated islets localized under the capsule (sections from two glands). After application of the double-cannula technique using 18-G + 22-G cannulas, the intraglandular location of islets was observed with evident surrounding hematoma (**middle** row, sections from one gland). After application of the double-cannula technique using 20-G + 24-G cannulas, the intraglandular location of islets was observed with occasional minimal hematoma (**lowermost** row, sections from one gland). Black arrowhead: location of the capsule, white arrow: intraglandular hematoma, and black asterisk: pancreatic islet mass. The brown color in the uppermost row represents positive staining.

We examined several techniques for islet implantation into the submandibular gland. These techniques included subcapsular implantation, direct injection into the gland with a 25-G butterfly needle (in 50–100 µL medium), and our double-cannula technique. According to the early histological assessment of islet integrity and localization, we decided to use the double-cannula technique as it showed the most promising preliminary results, especially regarding the location of the islets inside the SMG (Figure 1). This approach was technically feasible and was only occasionally associated with small intra-glandular hematoma. In this case, another site was used for transplantation.

In addition, we attempted to perform transductal islet transplantation through Wharton’s duct using the duct cannulation technique described by Kuriki et al. [13]. However, we found that the largest size of the cannula that could be safely introduced into the duct without injury was a 29-G cannula, which was not suitable because of the pancreatic islet size (50–500 µm in diameter). Therefore, this technique was abandoned.

### 3.2. Blood Glucose Measurements

Rats were considered cured if their blood glucose level was ≤200 mg/dL in two consecutive measurements. Two of twelve rats that received islet transplantation into the submandibular gland using the double-cannula technique showed the reversal of diabetes starting from day 12 after transplantation. The excision of the SMG in the cured rats resulted in the recurrence of diabetes, confirming the intraglandular graft function. On the other hand, transplanting the same amount of islets through the portal vein resulted in the reversal of diabetes in 4 of 6 rats. The graft also showed an earlier function in comparison to the SMG. In addition, one rat died on day two after transplantation in the IPO group; laparotomy showed intestinal gangrene.

### 3.3. IVGTT

An IVGTT was performed in rats that completed observation periods (SMG group, n = 4; IPO group, n = 3). The results are shown in “Supplementary Figure S2”. The AUC values in rats with intra-SMG grafts were significantly lower in comparison to diabetic rats (n = 3; *p* < 0.001) and significantly higher in comparison to normal Lewis rats (n = 6; *p* < 0.001). Similar findings were found in the case of IPO; the AUC values were significantly lower in comparison to diabetic rats (*p* < 0.001), while they were significantly higher in comparison to normal rats (*p* < 0.001). There was no significant difference between the SMG and IPO groups (*p* = 0.513).

### 3.4. Immunohistochemistry

**Figure 2:**
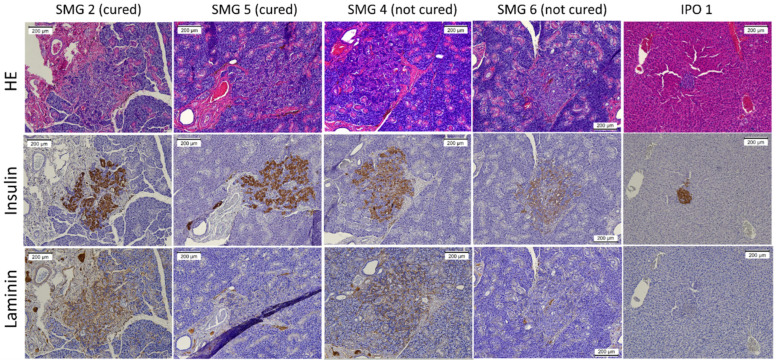
Immunohistochemistry of the SMG and IPO groups. Representative images showing HE, anti-insulin, and anti-laminin staining. The brown color represents positive staining.

The examination of early samples (four days after transplantation) showed that islets that are directly injected with a needle travel along the interlobular planes, finally reaching a subcapsular location. Interestingly, this was associated with islet disintegration and mononuclear cell infiltration. On the other hand, islets that were transplanted using the double-cannula method were found within the acinar tissue along the injection track formed by the cannula, commonly in an intact form. A histological examination revealed that the islets were surrounded by a varying degree of hematoma and localized inflammation. The histological examination of the glands after 31 days showed insulin-positive cells in all examined specimens (Figure 2). However, stronger insulin staining was observed in the samples of cured rats. Surviving islets in the SMG group had a large islet mass pattern in comparison to single islets in the IPO group. Of particular note, the surviving islet masses in the cured SMG group were frequently found to be connected to a major ductular system. Laminin staining showed variable patterns among the examined samples.

## 4. Discussion

Five studies previously investigated the SMG as a site for pancreatic islet transplantation in diabetic animal models, in addition to one morphological study in hamsters (Table 1).

In 1977, Georgakakis et al. [6] investigated the implantation of syngeneic islets into the SMG (n = 16, at four different sites) or pancreas (n = 16, at six different sites) of diabetic inbred adult albino Lewis rats. Rats were followed for two months. At three to four days after transplantation, blood glucose levels were nearly normal (200–250 mg/dL) in 13/16 rats in the pancreas group, while the SMG group showed only moderate amelioration towards normal values (around 300 mg/dL).

In 1992, Pour et al. [5] developed a model of pancreatic islet transplantation into the hamster SMG to study the role of islets in the initiation of pancreatic exocrine tumors induced by the carcinogen, N-nitrosobis (2-oxopropyl) amine (BOP). This hypothesis was based on the finding that BOP had no effect on the acinar or ductular cells of the SMG in comparison to the pancreas. In this study, homologous islets isolated from male Syrian hamsters were implanted into the left SMG through a 1 cm incision over the gland. The islets (200–400 islets in 100 µL) were injected using a 1 mL syringe and a 25-G needle that was inserted horizontally deep into the gland, and the islets were injected along the needle track during withdrawal. In eight out of ten hamsters, isolated islets or groups of well-preserved islets were found either embedded within acinar tissue (with well-defined margins) or close to large or small ducts (some showing connection with ductal walls). Failure in two out of ten hamsters was explained by the number of islets or the possibility of islet leakage through the needle track. Another study then used this model to demonstrate the role of islets in pancreatic carcinogenesis [14]. In addition, the ability of this model to reverse diabetes was investigated in two other studies in 2012. In the first study [15], eight diabetic male hamsters received homologous islets (200–400 in 100 µL of HBSS) implanted in the left SMG. Hamsters showed normal blood glucose levels starting from two weeks after transplantation until the end of the study (98–130 mg/dL). In the second study [16], 15 female hamsters received 750 freshly isolated homologous islets transplanted into the left SMG three days after the induction of diabetes. Ten of these hamsters had normal blood glucose levels at 12 weeks (96–125 mg/dL) while their pancreatic islets remained atrophic.

In 2011, Sandberg et al. [17] transplanted syngeneic islets (isolated from male C57BL/6 mice) into male diabetic recipients. The animals were observed for 12 days after transplantation and then sacrificed. The islets were collected in a braking pipette (volume 50–100 µL) and implanted under either the Lt renal capsule (300 islets [n = 7]) or by slow injection after puncture of the capsule of the right SMG gland (300 islets [n = 15] or 500 islets [n = 5]). Five of the seven showed normoglycemia starting one day after transplantation under the kidney capsule (two animals died of hypoglycemia). On the other hand, only two out of fifteen mice that received 300 islets and one out of five mice that received 500 islets in the SMG showed normoglycemia. In the histological examination, well-preserved islets and insulin-containing β-cells were only observed in the cured animals.

According to these studies, it seems that the successful correction of hyperglycemia that was achieved by Pour et al. after islet transplantation into the hamster SMG could not be reproduced in other murine models, including rat and mouse models. Based on our pilot study and morphological assessment, we assumed that this is a result of the failure of islets to reach a true intraparenchymal location with close association to the ductular and vascular system. Although we tried an injection technique such as that proposed by Pour et al. in hamsters, we found that in the rat model, the islets ended up dissecting their way into the subcapsular location and eventually degenerating. Therefore, we developed the double-cannula technique to ensure that the islets, with minimal-to-no fluid components, are directly ejected into an intraparenchymal location. By doing this, we could achieve several morphological features that were described by Pour et al. [15,16] in the hamster model. A similar pattern of delayed correction of hyperglycemia was also noted in the cured rats (glycemic improvement starting around day 12 after transplantation). However, the results of our technique were largely variable. Our pilot study also confirmed the low potential of transplantation into the SMG subcapsular location to cure diabetes in rats, as previously reported by Sandberg et al. [17].

In our experience, the tightly packed acinar tissue of the submandibular gland in the Lewis rat prevented the direct placement of pancreatic islets by needle injection. As a result, the development of a space for the pancreatic islets was necessary and was successfully achieved using our refined method. However, one main drawback of this method is the traumatic injury to the gland parenchyma. Although it is technically feasible to avoid the main ductular system of the gland during the introduction of the cannula, the degree of parenchymal trauma and subsequent degree of inflammation could not be predicted. This might explain the variability of the islet function observed in our study. Laminin, an extracellular matrix (ECM) component, was shown to positively influence islet function [18]. We hypothesized that the submandibular gland could provide suitable ECM components for optimal islet function due to its anatomic and histologic similarity to the pancreas. Although laminin was detected around the islet region in some samples, it was neither a constant finding nor specific to samples from rats that achieved a cure.

In conclusion, our results showed that the transplantation of pancreatic islets into the submandibular gland is feasible and that the tissue environment of the SMG, which is composed of acinar and ductular tissue, can maintain the viability and function of the islet; however, it showed remarkable variability. Using our technique, we could consistently place the islets within the SMG acinar tissue, rendering it an attractive technique to study islet–acinar cell interaction and for in vivo stem cell differentiation studies. Further refinement of the technique is required to obtain tight glycemic control and avoid adverse inflammatory outcomes. Transplantation into the rat SMG showed no advantage over the conventional approach of intra-portal transplantation.

**Table 1:** Studies that investigated the submandibular gland as a site for pancreatic islet transplantation.

Study	Islets Isolation	Recipients	Technique	Compara-tive Group	Study Duration	Compl-ications	Blood Glucose Level	Histology
Gero-gakakis, et al., 1977 [6]	Modified collagenase digestion, freshly isolated	16 Adult inbred albino Lewis rats (200–400 g), diabetic by STZ	Direct injection into 4 different sites (200–400 islets)	Normal control, Diabetic control, pancreatic injection.	2 months	SMG abscess in 2 rats	Relative improvement of weight and BGL (around 300 mg/dl). BGL rose slightly again after 1 month.	Intact and viable islets within SMG.
Pour, et al., 1992 [5]	Modified collagenase digestion, freshly isolated	10 syngeneic male Syrian hamsters, non-diabetic.	Using 1-mL syringe & 25-G needle inserted horizontally deep into the gland after 1-cm incision (200–400 islets in 100 µL HBSS)	None	6 weeks	None	Not measured (morphologic study)	8/10 showed well-preserved islets, good vascularization, few inflammatory cells, positivity for β, α, and δ cells. Normal pancreas
Sand-berg, et al., 2011 [17]	Collagenase digestion, cultured in RPMI for 3–4 days.	Male C57BL/6 mice, diabetic by alloxan.	Islets collected in braking pipette. Slow injection after capsule puncture (Rt): 300 islets, n = 15.500 islets, n = 5	Under Lt renal capsule, 300 islets, n = 7.	12 days	2/15 & 1/5 died due to unknown reasons	Normoglycemia in 2/15 (300 islets group) & 1/5 (500 islets group).	Well-preserved & insulin-positive in cured animals. Occasional weak insulin stain in non-cured mice.
Pour, et al., 2012 [15]	Modified collagenase technique.	10 syngeneic male hamsters. Diabetic by STZ.	Direct injection into Lt SMG, 200–400 islets in 100 µL HBSS.	None	8 weeks	2 died of hyperglycemia.	8/10 showed normoglycemia after 2 weeks (98–130 mg/dL).	Well-preserved multiple islets foci. positivity for β, α, δ, and a few PP cells. 10–30× higher labeling index than normal islets.
Pour, et al., 2012 [16]	Modified collagenase digestion.	15 syngeneic female Syrian hamsters (8 weeks old). Diabetic by STZ.	Direct injection deep into Lt SMG using 1-mL Hamilton syringe, 25-cm PE-50 tube & 26-G needle.	None	12 weeks	None	Measured before Tx & at termination only. 10/15 became normoglycemic (96–125 mg/dL)	Well-preserved islet clusters in SMG in 13/15. Atrophic pancreas if normoglycemic (10/15)
Current study	Modified collagenase digestion	12 syngeneic male Lewis rats (8–10 weeks old). Diabetic by STZ.	Double-cannula technique	Intra-portal tx (n = 6)	31 days	None	2/12 showed normoglycemia	Well-preserved & insulin-positive in cured rats. Weak insulin stain in non-cured rats

BGL, blood glucose level; gl, glucose; Lt, left; Rt, right; SMG, submandibular gland; STZ, streptozotocin; Tx, transplantation.

## Data Availability

The data supporting the reported results are supplied in the manuscript and the Supplementary File.

## Supplementary Materials

The following supporting information can be downloaded at: https://www.mdpi.com/article/10.3390/jcm12113735/s1, Excel File: Datasets for blood glucose levels and IVGTT results in SMG and IPO groups; Supplementary Figure S1: Illustration of the salivary gland anatomy in a Lewis rat; Supplementary Figure S2: Intravenous glucose tolerance test (IVGTT) results.
