# Supplementary material for: Pancreatic Islet Transplantation into the Submandibular Gland: Our Experimental Experience and a Review of the Relevant Literature
Source: J Clin Med. 2023 May 29;12(11):3735. doi: 10.3390/jcm12113735 (PMC10253336; doi:10.3390/jcm12113735)
Supplement: Supplementary file 1 [file jcm-12-03735-s001.zip › jcm-2321822-Supplementary figures.pdf]

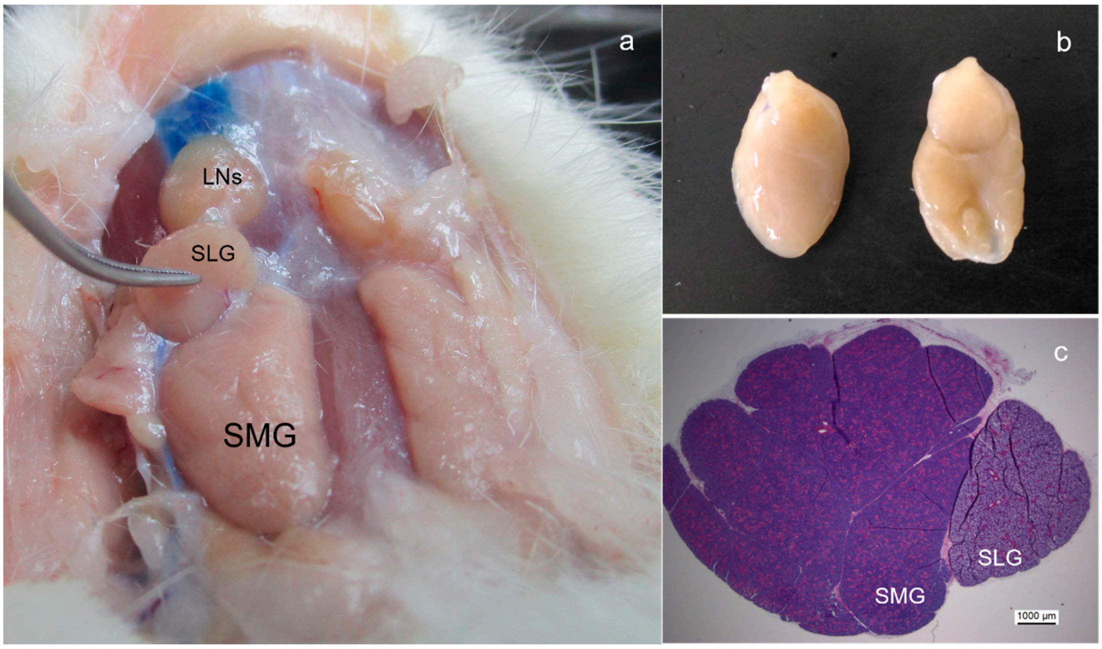

Figure S1. Illustration of the salivary gland anatomy in a Lewis rat. Cadaveric dissection (a), gross morphology (b), histology of the normal submandibular and sublingual glands (c). LNs, lymph nodes; SLG, sublingual gland; SMG, submandibular gland.

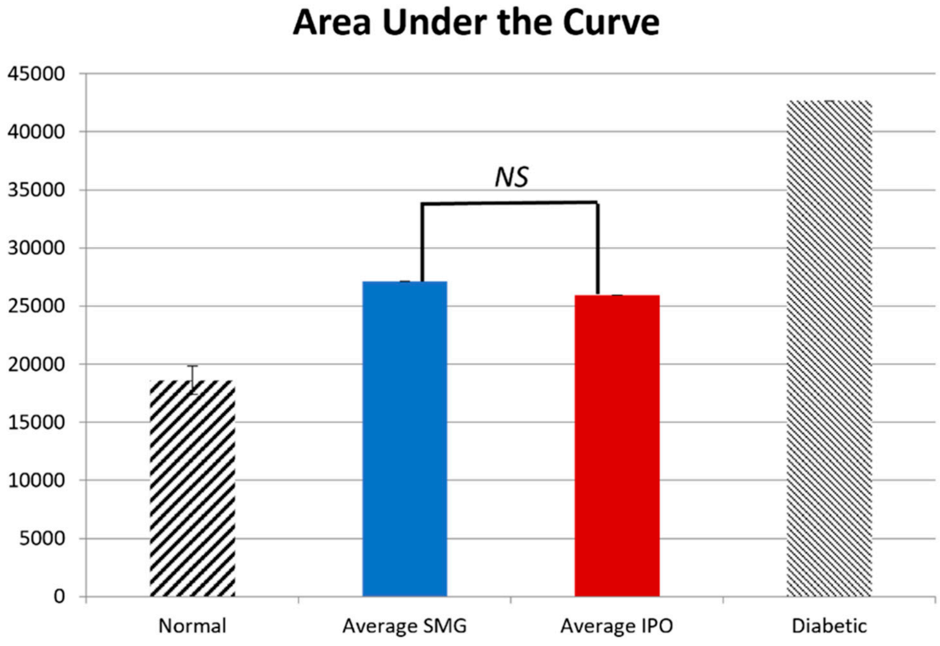

Figure S2. Intravenous glucose tolerance test (IVGTT) results. The average values of the Submandibular gland and Intraportal groups were compared to those of normal and diabetic rats. NS, not significant.
